# One- and two-stage surgical revision of infected shoulder prostheses following arthroplasty surgery: A systematic review and meta-analysis

**DOI:** 10.1038/s41598-018-36313-3

**Published:** 2019-01-18

**Authors:** Setor K. Kunutsor, Vikki Wylde, Andrew D. Beswick, Michael R. Whitehouse, Ashley W. Blom

**Affiliations:** 1 National Institute for Health Research Bristol Biomedical Research Centre, University Hospitals Bristol NHS Foundation Trust and University of Bristol, Bristol, UK; 2 Translational Health Sciences, Bristol Medical School, Musculoskeletal Research Unit, University of Bristol, Learning & Research Building (Level 1), Southmead Hospital, Bristol, BS10 5NB UK

## Abstract

Periprosthetic joint infection (PJI) is a catastrophic complication of shoulder arthroplasty. Commonly used surgical treatments include one- or two-stage revision, but their effectiveness in controlling infection is uncertain. We aimed to compare re-infection (recurrent and new infections) rates; clinical measures of function and pain; and noninfection complication rates of one- and two-stage revision surgery for shoulder PJI using a systematic review and meta-analysis. We searched MEDLINE, Embase, Web of Science, and The Cochrane Library to February 2018. Longitudinal studies conducted in patients with shoulder PJI treated exclusively by one- or two-stage revision were eligible. No clinical trials were identified. Re-infection rates were meta-analysed using random-effect models after arcsine transformation. The re-infection rate (95% CI) in pooled analysis of eight one-stage studies (147 participants) was 5.3% (1.4–10.6). The corresponding rate for 27 two-stage studies (351 participants) was 11.5% (6.0–18.1). Postoperative clinical measures of function and pain were not significantly different between the two revision strategies. The pooled noninfection complication rate (95% CI) for one-stage and two-stage revision was 12.1% (6.1–19.5) and 18.9% (8.4–31.9) respectively. New evidence suggests one-stage revision is at least equally as effective as the two-stage in controlling infection, maintaining joint function, and improving complications in shoulder PJI.

## Introduction

For many people with severe gleno-humeral disease, fracture, avascular necrosis or rotator cuff wear, shoulder arthroplasty is considered the most effective surgical intervention for alleviating pain and disability. In the UK in 2016, about 7000 shoulder arthroplasties were performed^1^ and across Europe, procedures are more common with a six-fold higher rate in Germany^2^. As with periprosthetic joint infection (PJI) of the hip and knee, deep PJI following primary anatomic total shoulder arthroplasty (TSA) is a catastrophic complication which affects between 0.4 to 2.9% of patients^3–5^. The incidence of infection in primary reverse total shoulder arthroplasty (RSA) is about six times higher compared with that of primary anatomic TSA^6^. Treatment of shoulder PJI is a challenging task with the key aims of infection control, pain relief, and restoration of joint function. There is considerable controversy as to the optimal treatment strategy to achieve these aims. Treatment options for PJIs following shoulder arthroplasty are similar to those for hip and knee PJI and they include: long-term suppressive antibiotic treatment without surgical intervention; debridement, treatment with antibiotics and retention of the prosthesis (DAIR); one-stage and two-stage revision; and resection arthroplasty. Choice of treatment strategy is largely based on the experiences of treating surgeons and data from hip and knee arthroplasty studies^7^. Though the treatment option depends on the chronicity of infection, organism isolated and its virulence, component stability and patient fitness for surgery^8^, the one- and two-stage revision strategies are preferred as they are associated with successful clearance of infection and good functional outcomes^9,10^. As with PJIs after hip and knee arthroplasty, the two-stage revision strategy is regarded as the standard treatment option for shoulder PJI because it is generally consistently associated with good rates of infection control^10–12^. However, this revision strategy may be associated with functional impairment; though some studies have reported improved shoulder function after two-stage revision^10,12^. The one-stage is emerging as a promising alternative option; however, it is not as popular as the two-stage strategy. With regards to infection control, the evidence has been inconsistent for one-stage revision. Whiles some series have reported good infection control^9,13^, a high risk of infection recurrence has also been reported^14^. Emerging data suggests that one- and two-stage revision strategies for hip and knee PJI are comparable in terms of infection control^15,16^. No randomised controlled trial (RCT) has compared the effectiveness of the one- and two-stage revision procedures for managing shoulder PJI. However, a number of studies have reviewed the existing evidence by comparing results of case series that have reported findings on any of these two revision strategies and the results have been mostly inconclusive. A qualitative and quantitative review of 30 studies that compared infection control and functional outcomes among treatment options for shoulder PJI concluded that the one-stage may be as effective as the two-stage revision strategy, though infection control was reported to be better with two-stage revision^17^. However, one-stage revision produced a higher mean Constant-Murley (CM) score compared with that of two-stage revision. In another review which was based on six one-stage and 13 two-stage studies, George and colleagues reported a higher infection eradication rate for one-stage revision compared with two-stage revision^18^. In a recent overview study of existing evidence, the authors concluded that two-stage revision is the recommended strategy for PJI of the shoulder, but that a one-stage strategy can be used if the infecting organism is identified pre-operatively^3^.

The current evidence suggests that the optimal treatment strategy for the management of PJI of the shoulder is uncertain. There were several features of these previous reviews which limited the generalisability of the findings. First, these reviews did not pool the evidence using appropriate meta-analytic methods, which should take into account the heterogeneity of the included studies. Second, because the reviews did not pool the evidence using standard techniques, potential sources of heterogeneity among the contributing studies were not explored and no subgroup analysis was conducted across relevant characteristics. Third, sources of biases such as heterogeneity and preferential publication bias were not assessed. Finally, several new individual studies have been published recently. In this context, using a systematic review and meta-analysis, we aimed to conduct a detailed and robust comparison of the effectiveness of the one- and two-stages revision strategies for shoulder PJI using re-infection as a primary outcome and under a range of study-level clinical characteristics. Secondary objectives included (i) comparing the effectiveness of the one- and two-stage revision strategies using other clinical outcomes such as measures of function, pain, and satisfaction as well as noninfection-related complication rates and (ii) to explore for potential sources of heterogeneity between studies and assess publication bias.

## Methods

### Data sources and search strategy

The review was registered in the PROSPERO prospective register of systematic reviews (CRD42017082747) and it was conducted based on a predefined protocol and in accordance with PRISMA and MOOSE guidelines^19,20^ (Appendix 1 and 2). We searched for longitudinal studies (observational retrospective and prospective cohort studies, case cohort studies, nested case control studies, or RCTs) reporting re-infection events and/or other clinical outcomes following one- or two-stage surgical revision of infected shoulder prostheses in the following databases: MEDLINE, Embase, Web of Science, and The Cochrane library from inception up to 10 February 2018. The computer-based searches combined free and MeSH search terms and combination of key words related to the intervention (e.g., “one-stage revision”, “two-stage revision”), population (“shoulder arthroplasty”) and PJI (e.g., “prosthetic joint infection”, “deep infection”, “surgical site infection”). There were no language restrictions. The search was complemented by manually scanning reference lists of identified relevant articles and review articles on the topic for publications missed by the original search. Further details on the search strategy are presented in Appendix 3.

### Eligibility criteria

We included studies that reported recruiting patients with PJI following shoulder arthroplasty (anatomic TSA and/or RSA) and who were treated exclusively by a one-stage or two-stage revision strategy, and followed post-operatively for re-infection (defined as recurrence of infection by the same organism(s) and/or re-infection with a new organism(s)) and/or other clinical outcomes such as (i) function [as measured by CM score (a commonly used functional score for the shoulder that assesses pain, activities of daily living, range of movement, and strength)^21^, American Shoulder and Elbow Surgeons (ASES) Shoulder Assessment score, Simple Shoulder Test (SST), University of California Los Angeles (UCLA) score (function component), Disabilities of the Arm Shoulder and Hand score (DASH), Penn Shoulder Score (function component), forward flexion, abduction, external rotation, and range of motion]; (ii) pain [as measured by pain scores, visual analogue scores (VAS), Penn Shoulder Score (pain component), UCLA score (pain component)]; (iii) satisfaction [as measured by Penn Shoulder Score (satisfaction component)]; and noninfection-related complications (such as dislocation, fracture, loosening, haematoma, postoperative instability, radial nerve entrapment, non-union, and arthrofibrosis).

### Data extraction and quality assessment

After the initial screen of titles and abstracts by one reviewer (S.K.K.), we acquired potentially relevant articles for detailed full text evaluation. Two independent reviewers (S.K.K., V.W.) assessed each article using the inclusion criteria and any disagreements regarding eligibility of an article was discussed, and consensus reached with a third author (A.D.B.). The data was independently extracted by one reviewer (S.K.K.) using a standardized data collection and quality assessments were also conducted. A second reviewer (V.W.) checked these data with that in original articles. We extracted data on year of publication, study design, country and geographical location (continent), mean/median baseline age, proportion of males, type of index arthroplasty, type of revision surgery, use and type of spacer, revision surgery characteristics, period of follow-up after revision surgery, number of re-infection outcomes and participants, and measures of pain, function, and satisfaction. For multiple publications involving the same cohort or series, the study with the most comprehensive information was used. We also corresponded with study investigators to provide missing information where relevant. We assessed the methodological quality of included studies based on the Methodological Index for Non-Randomised Studies (MINORS), a validated instrument which is designed for assessment of the quality of non-randomised studies in surgery^22^ and has been described in previous reports^15,16^. This tool uses eight pre-defined domains namely: a clearly stated aim, inclusion of consecutive patients, prospective collection of data, endpoints appropriate to the aim of the study, unbiased assessment of the study endpoint, follow-up period appropriate to the aim of the study, loss to follow-up less than 5%, and prospective calculation of the study size. For each item, the instrument assigns a score of 0 for “not reported”, 1 for “reported but inadequate”, or 2 for “reported and adequate”. These are then summed up into a total score. The global ideal score is 16.

### Statistical analysis

The rate of re-infection (estimated from the number of re-infections within follow-up period after shoulder revision surgery/total number of participants with PJI or number of shoulder joints with PJI) with 95% confidence intervals (CIs) was used as the summary measure across studies. Rates were estimated using the Freeman-Tukey variance stabilising double arcsine transformation^23^ because of the use of binary data with low rates. Details of the method have been reported in previous reports^15,16^. Summary rates of re-infection were pooled using random effects models to account for the effect of between-study heterogeneity^24^. Quantification of the extent of statistical heterogeneity across studies employed standard chi-square tests and the I^2^ statistic^25^. Potential sources of heterogeneity by study-level and clinically relevant characteristics were explored using stratified analysis and meta-regression^26^. Publication bias was assessed using Egger’s regression symmetry test^27^. Noninfection-related complication rates (estimated from the number of complications within follow-up period after shoulder revision surgery/total number of participants with PJI or number of shoulder joints with PJI) with 95% CIs were also estimated. Other clinical measures of function and pain were compared between the two revision strategies using descriptive statistics (Wilcoxon rank-sum tests). We employed Stata version 14 (Stata Corp, College Station, Texas, USA) for all statistical analyses.

## Results

### Study identification and selection

**Figure 1:**
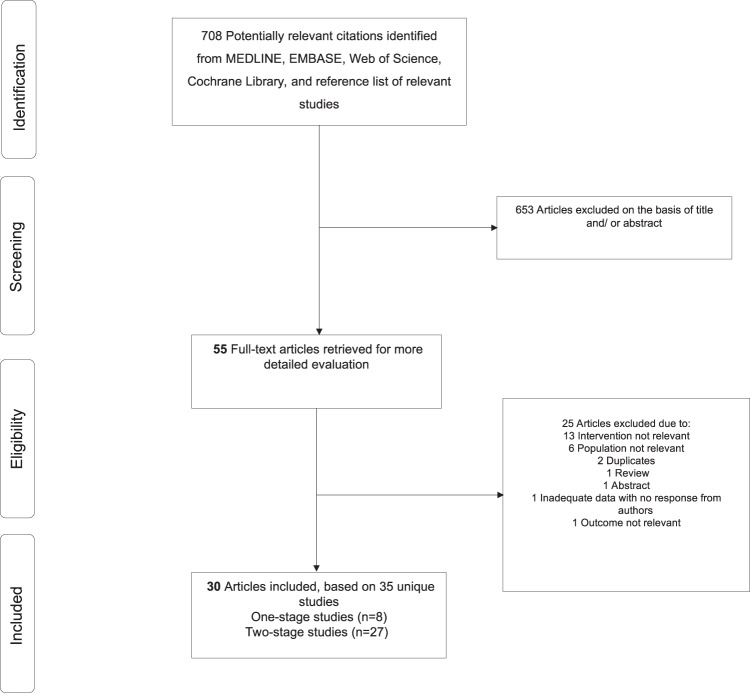
PRISMA flow diagram.

The computer search and manual screening of reference lists of relevant studies identified 708 potentially relevant citations. After exclusions based on titles and abstracts, 55 articles remained for detailed evaluation. The remaining 30 articles were pertinent to the review question and were included in the pooled analysis (Fig. 1; Tables 1 and 2; Appendix 4). Overall, there were 35 unique study populations (comprising of 498 participants or shoulders revised for PJI and 69 re-infections) eligible for the review.

**Table 1:** Summary characteristics of included studies.

	One-stage revision	Two-stage revision	*P*-value
**Eligible studies**			
Total number of studies included	8	27	
**Participants**			
Total number of participants	147	351	
Total number of re-infections	12	57	
Median (IQR) age (years)	66.3 (64.0–67.3)	63.0 (62.0–65.0)	0.31
Median (IQR) males (%)	68.3 (57.2–79.2)	62.5 (41.7–73.5)	0.32
**Location, number of studies (number of participants)**			
Europe	6 (85)	11 (131)	
North America	2 (62)	15 (208)	
Asia	0 (0)	1 (12)	
**Study and surgery characteristics**			
Median (IQR) time from index surgery to infection diagnosis (months)	4.0 (4.0–4.0)	25.2 (8.1–40.2)	0.48
Median (IQR) time from index surgery to revision surgery (months)	15.2 (15.2–15.2)	24.0 (8.0–40.0)	>0.99
Median (IQR) from infection diagnosis to revision surgery (months)	28.0 (28.0–28.0)	9.5 (2.5–14.0)	0.18
Median (IQR) interval between stages (months)	—	4.0 (3.0–6.6)	
Median (IQR) follow-up (years)	3.0 (3.0–3.8)	3.9 (3.0–4.5)	0.36
Methodological quality (IQR)	10 (10–11)	10 (10–11)	0.77
**Baseline clinical characteristics**			
Median (IQR) Constant-Murley score	31.5 (27.5–37.4)	29.0 (21.0–34.5)	0.47
Median (IQR) ASES total score	33.6 (33.6–33.6)	32.0 (31.7–32.3)	0.22
Median (IQR) ASES function score	10.6 (10.6–10.6)	11.2 (11.2–11.2)	0.32
Median (IQR) SST score	1.8 (1.8–1.8)	1.8 (1.8–1.8)	NE
Median (IQR) Forward flexion	57.0 (57.0–57.0)	58.0 (45.0–60.0)	0.77
Median (IQR) External rotation	23.0 (23.0–23.0)	14.0 (13.0–14.0)	0.37
Median (IQR) Internal rotation	3.0 (3.0–3.0)	2.0 (2.0–2.0)	0.32
Median (IQR) Abduction	46.0 (46.0–46.0)	51.0 (42.0–60.0)	>0.99
Median (IQR) Penn shoulder total score	—	24.9 (24.9–24.9)	NE
Median (IQR) ASES pain score	24.2 (24.2–24.2)	20.5 (20.5–20.5)	0.32
Median (IQR) Pain score*	—	4.2 (4.2–4.4)	—
Median (IQR) VAS score	—	7.08 (7.08–7.08)	—

ASES, American Shoulder and Elbow Surgeons; IQR, interquartile range; NE, not estimated; SST, Simple Shoulder Test; VAS, visual analog scale.

*None of the one-stage revision studies reported scores for these clinical characteristics.

**Table 2:** Characteristics of studies included in review.

Lead Author, Publication Date (Reference No.)	Location	Year of study	Mean/median age (years)	% male	Mean/median interval between stages (months)	Type of index arthroplasty	Common infecting organism (s)	Follow up Mean/median (years)	No. of re-infections	No. of noninfection complications	No. of participants or shoulders	Quality score
**One-stage**												
Coste, 2004	France	1991–1999	NR	NR	NA	NR	*S. epidermidis, S. albus, C. acnes*	2.7*	0	NR	3	10
Beekman, 2010	Belgium	2005–2007	62.0	81.8	NA	RSA	*C. acnes*	2.0	1	3	11	11
Grosso, 2012	USA	2001–2009	66.5	76.5	NA	Mix	*C. acnes*	3.0	1	1	17	11
Amaravathi, 2012	France	1993–2008	NR	NR	NA	NR	*S. epidermidis*	2.5*	4	NR	12	10
Klatte, 2013	Germany	1990–2010	66.0	54.3	NA	Mix	*S. epidermidis*	4.7	2	5	35	8
Middernacht, 2014	Belgium	2004–2009	NR	NR	NA	RSA	*C. acnes*	3.4*	2	NR	19	12
Jacquot, 2015	France	1996–2011	NR	NR	NA	RSA	*C. acnes*	3.0*	0	NR	5	10
Stone, 2017	USA	2004–2012	68.0	60.0	NA	NR	*C. acnes*	3.8*	2	5	45	10
**Two-stage**												
Sperling, 2001	USA	1972–1994	NR	NR	6.6	NR	Pseudomonas diminuta, CNSA, SA	4.8	0	NR	3	10
Seitz, 2002	USA	NR	62.0*	62.5*	NR	NR	S. aureus*	4.8*	0	NR	5	9
Jerosch, 2003	Germany	NR	71.0*	NR	NR	NR	*S. aureus*	0.5–2.5*	0	NR	10	9
Coste, 2004	France	1991–1999	NR	NR	NR	NR	*S. epidermidis*	2.7*	4	NR	10	10
Mileti, 2004	USA	1975–2000	58.0	25.0	NR	Mix	*S. epidermidis*	7.4	0	NR	4	10
Dines, 2006	USA	NR	NR	NR	NR	NR	NR	6.3*	0	NR	3	10
Strickland, 2008	USA	1995–2004	62.0	58.8	2.5	NR	*C. acnes* or CNSA	2.9	7	14	19	10
Kelly, 2009	USA	2005–2007	NR	NR	NR	Mix	*C. acnes*	1.8*	2	NR	8	9
Dodson, 2010	USA	2002–2006	NR	NR	NR	NR	*C. acnes*	4.0*	2	NR	5	10
Hattrup, 2010	USA	1997–2005	NR	NR	6.6	NR	CNSA	4.1	3	NR	20	9
Stine, 2010	USA	2003–2007	NR	NR	NR	NR	NR	2.3	0	3	12	10
Jawa, 2011	USA	2000–2006	63.0	71.4	8.9	Mix	*C. acnes*	2.3	5	4	28	11
Sabesan, 2011	USA	2001–2009	67.6	58.8	4.0	Mix	CNSA	3.9	1	5	17	11
Weber, 2011	Germany	1998–2008	62.5	50.0	NR	NR	NR	4.0*	0	NR	4	11
Amaravathi, 2012	France	1993–2008	NR	NR	NR	NR	*C. acnes*	2.5*	6	NR	12	10
Romano, 2012	Multiple countries in Europe	1999–2009	NR	NR	NR	Mix	MRSA	3.4*	0	NR	17	11
Achermann, 2013	Switzerland	1998–2010	61.6	16.6	3.0	Anatomic and RSA	*S. species*	3.8	1	NR	7	10
Ghijselings, 2013	Belgium	2001–2012	65.0	33.3	3.2	Mix	NR	4.7	0	1	3	10
Ortmaier, 2014	Austria	1998–2010	NR	75.0	≥3.0	RSA	NR	>2.0*	3	2	12	11
Middernacht, 2014	Belgium	2004–2009	NR	NR	NR	RSA	C. acnes*	3.4*	1	NR	4	12
Jacquot, 2015	France	1996–2011	NR	NR	NR	RSA	*C. acnes*	3.0*	5	NR	14	9
Zhang, 2015	USA	2005–2012	69.0*	83.3*	NR	Mix	*C. acnes*	2.0*	0	NR	11	10
Assenmacher, 2017	USA	1980–2010	65.0	73.5	2.5	Mix	*C. acnes*	4.1	6	2	35	12
Buchalter, 2017	USA	2000–2014	63.0	74.0	4.9	Mix	*C. acnes*	5.3	5	3	19	11
Lee, 2017	Korea	2009–2014	69.5	41.7	NR	Mix	*MRSA*	3.4	0	2	12	10
Stone, 2017	USA	2004–2012	65.0	68.0	NR	NR	*C. acnes*	3.8*	4	2	19	10
Grubhofer, 2018	Switzerland	2000–2013	62.0	63.2	7.0	Mix	CNSA	4.3	2	2	38	11

C, *Cutibacterium*; CNSA, Coagulase negative *Staphylococcus aureus*; MRSA, Methicillin resistant *Staphylococcus aureus*; NA, not applicable; NR, not reported; RSA, reverse total shoulder arthroplasty; S, Staphylococcus; *for the entire sample; Mix, mixture of hemiarthroplasty, anatomic total shoulder arthroplasty, and reverse total shoulder arthroplasty.

### Study characteristics and study quality

Table 1 summarises baseline characteristics of one- and two-stage revision studies included in the review and Table 2 provides baseline characteristics and quality assessment scores of the individual studies. There were no significant differences in baseline study level surgery and clinical characteristics between the two revision strategies. All included studies were based on retrospective analyses of observational cohort data. No clinical trials comparing both revision strategies were identified. *Cutibacterium acnes* (*C. acnes*) was the most commonly causative organism for PJI of the shoulder in the majority of eligible studies. Studies were carried out in Europe (Austria, Belgium, France, Germany, and Switzerland), North America (United States of America), and Asia (South Korea). The methodological quality of included studies ranged from 8–12.

### One-stage revision and re-infection

**Figure 2:**
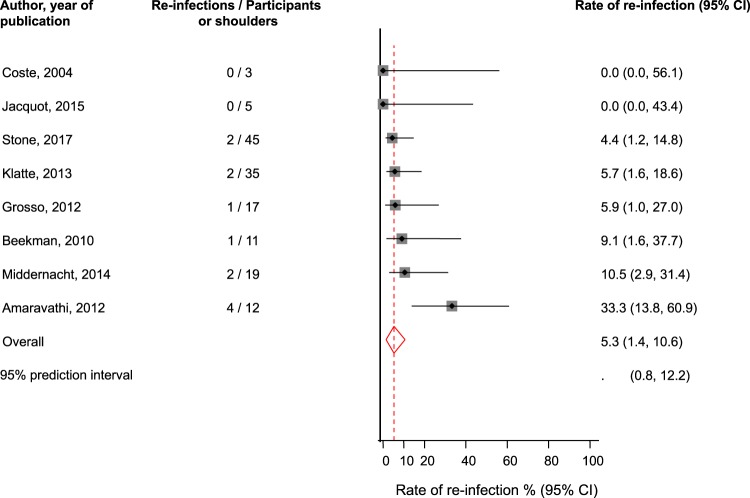
Rates of re-infection in patients treated by one-stage revision. The summary estimates presented were calculated using random effects models; CI, confidence interval (bars).

Eight studies comprising of 147 participants evaluated the one-stage revision strategy and recorded 12 re-infections on follow-up (Tables 1 and 2). The pooled random effects re-infection rate (95% CI) was 5.3% (1.4–10.6; *p* < 0.001) (Fig. 2). The 95% prediction interval for the pooled re-infection rate was 0.8 to 12.2%, suggesting that the true re-infection rate for any single new study will usually fall within this range. There was no evidence of heterogeneity between contributing studies (*I*^2^ = 0%, 95% CI: 0–68%; *p* = 0.40). There was no statistically significant evidence of publication bias using the Egger test (*p* = 0.66).

### Two-stage revision and re-infection

**Figure 3:**
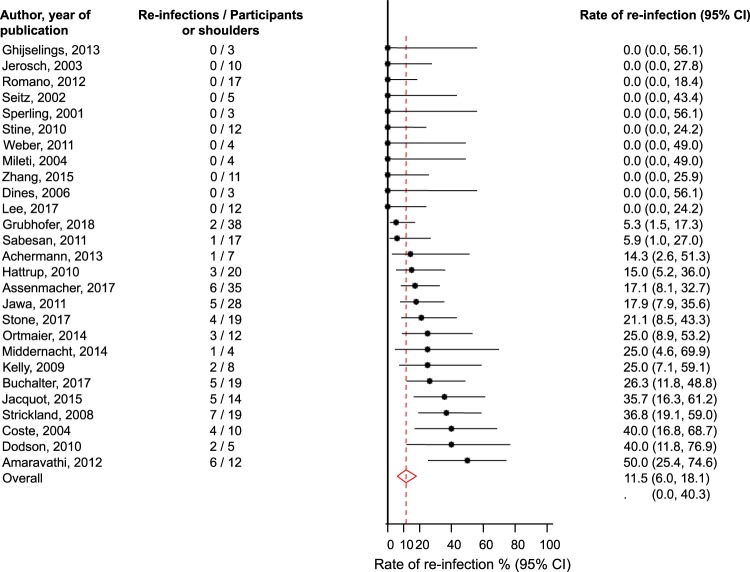
Rate of re-infection in patients treated by two-stage revision. The summary estimates presented were calculated using random effects models; CI, confidence interval (bars).

Twenty-seven studies comprising of 351 participants with PJI of the shoulder reported 57 re-infections following two-stage surgical revision (Tables 1 and 2). The pooled re-infection rate (95% CI) was 11.5% (6.0–18.1; *p* < 0.001) with a 95% prediction interval of 0.0 to 40.3% (Fig. 3). There was moderate heterogeneity between contributing studies (*I*^2^ = 50%, 22–68%; *p* < 0.01), which was not explained by any of the study-level characteristics assessed in subgroup analyses (Appendix 5). Heterogeneity was substantially reduced (*I*^2^ = 41%, 0–73%; *p* = 0.10), when we restricted the analysis to studies of the highest quality (≥11). Among the higher quality studies, the pooled re-infection rate (95% CI) was 10.4 (4.0–18.5; *P* < 0.001), which was similar to the main finding. There was no evidence of publication bias (Egger’s *p* = 0.71).

In a subgroup analysis that compared re-infection rates between the two revision strategies, no significant difference was observed (*p*-value for meta-regression = 0.41).

### Other post-operative clinical outcomes

Fifteen studies (4 one-stage and 11 two-stage studies) reported on complications following revision surgery and these included dislocations; humeral or clavicular fracture; humeral or glenoid loosening; haematoma; postoperative instability; radial nerve entrapment and palsy; non-union; glenoid baseplate failure; glenosphere dissociation; and arthrofibrosis (Table 2). The pooled noninfection complication rates (95% CIs) for one-stage and two-stage revision were 12.1% (6.1–19.5; *p* < 0.001) and 18.9% (8.4–31.9; *p* < 0.001) respectively. In meta-regression analysis, no significant difference was observed (*p*-value for meta-regression = 0.62).

There were no significant differences in measures of function and pain between both revision strategies (Table 3). However, the scores for CM, forward flexion and abduction were better in the one-stage revision group compared with two-stage stage revision group; whereas scores for ASES, SST, external rotation, internal rotation, and Penn Shoulder were better in the two-stage revision group. Data on measures of satisfaction was very limited and could not be compared.

**Table 3:** Post-operative clinical outcomes following one- and two-stage revision strategies.

	One-stage revision	Two-stage revision	*P*-value
Median (IQR) Constant-Murley score	52.0 (51.6–53.0)	43.5 (37.6–58.1)	0.24
Median (IQR) ASES total score	60.0 (60.0–60.0)	68.2 (65.8–70.0)	0.16
Median (IQR) ASES function score	24.2 (24.2–24.2)	27.4 (27.4–27.4)	0.32
Median (IQR) SST score	5.0 (5.0–5.0)	5.6 (3.2–6.3)	0.72
Median (IQR) Forward flexion	120.5 (113.0–127.9)	95.0 (80.0–121.0)	0.20
Median (IQR) External rotation	23.0 (14.0–32.0)	36.0 (26.0–41.0)	0.13
Median (IQR) Internal rotation	4.0 (4.0–4.0)	9.9 (3.0–16.7)	>0.99
Median (IQR) Abduction	102.0 (102.0–102.0)	83.0 (80.0–116.0)	0.77
Median (IQR) Penn shoulder total score	50.6 (50.6–50.6)	67.4 (66.4–68.4)	0.22
Median (IQR) ASES pain score	39.6 (39.6–39.6)	40.0 (40.0–40.0)	0.32
Median (IQR) Pain score	—	2.0 (1.8–2.0)	—
Median (IQR) VAS score*	—	2.3 (1.3–5.0)	—

ASES, American Shoulder and Elbow Surgeons; IQR, interquartile range; Simple Shoulder Test; VAS, visual analog scale.

*None of the one-stage revision studies reported scores for these clinical characteristics.

## Discussion

### Key findings

We have compared the effectiveness of one- and two-stage revision strategies for PJI following shoulder arthroplasty using a systematic review and meta-analytic approach. Our primary outcome was re-infection (recurrent and new infections) with clinical measures of function and pain as well as noninfection complication rates employed as secondary outcomes. Pooled analysis showed that one-stage revision was associated with a markedly lower re-infection rate (5.3%) compared with the two-stage strategy (11.5%), though the difference was not statistically significant. Re-infection rates were generally similar across several study level clinically relevant characteristics for two-stage revision. The noninfection complication rate was also lower for one-stage revision compared with two-stage revision, though not significantly different. Though there were no significant differences in measures of function and pain between the two revision strategies, scores for CM, forward flexion, and abduction were better in one-stage revision patients; whereas scores for ASES, SST, external rotation, internal rotation, and Penn Shoulder were better in two-stage revision patients. However, given the limited data for these clinical outcomes, the findings should be interpreted with caution.

### Comparison with previous work

In this comprehensive quantitative review, we report several relevant findings that have not been previously reported. Some of our findings are also consistent with that of previous reviews on the topic. Consistent with our results, George and colleagues in their review of 20 studies reported that one-stage revision was associated with better infection control compared with two-stage revision and the difference was not significant^18^. Functional outcomes as measured by CM score was also better for the one-stage revision. In a systematic review including 15 studies, Marcheggiani and colleagues reported significantly better infection control rates for one-stage revision compared to two-stage revision, with no difference between CM scores^28^. Nelson and colleagues reported higher infection control for two-stage revision; however, in their systematic review including 30 studies, the difference between the two strategies was not significant^17^. The CM score for one-stage revision was also better but not significantly different. In our study, we employed a robust meta-analytical approach which took into account appropriate weighting of studies, whereas previous reviews simply summed the number of patients and number of infections, an approach which gives misleading results^29^. In addition to reporting data on noninfection complication rates and other measures of function as well as pain, we were careful not to include studies with overlapping participants; an approach that was ignored by the previous reviews. For example, the study of Klatte *et al*.^30^, included participants that had been included in the two studies of Ince *et al*.^13,31^. We also only included studies of patients with PJI following a shoulder arthroplasty as the index procedure and not other procedures such as rotator cuff surgery and internal fixation; as inclusion of these could have biased our results. In the absence of clinical trial data, our results provide up-to-date reliable evidence on the comparative effectiveness of the two revision strategies, as they are based on a larger number of studies and outcomes and employed a meta-analytic approach taking into account the heterogeneity between contributing studies.

### Implications of our findings

Overall, findings from our study suggest that the one-stage revision strategy for the management of PJI of the shoulder is at least equally as effective as the two-stage in terms of infection control and maintenance of joint function. Indeed, the principal aim of treatment is to eradicate and prevent recurrence of infection as well as optimise joint function^32^. Noninfection complications were also lower for one-stage revision. There are no clear management guidelines or consensus as to which revision strategy to use for PJI of the shoulder. Considerable research into the treatment of PJI has been carried out in patients with lower limb arthroplasty and this body of evidence has provided insight for treatment of shoulder infection^7^. However, infections in shoulder arthroplasty are characterised by different infective organisms, signs and symptoms, laboratory data, and also run a different clinical course^33^. The treatment of PJIs in the shoulder joint is indeed a challenging task for both the surgeon and the healthcare system. George *et al*. report that unlike PJI after hip and knee arthroplasty, the two-stage revision is not considered the “gold standard” for the management of PJI following shoulder arthroplasty, as there are other treatment options which report favourable outcomes^18^. However, the two-stage revision is generally considered as the standard treatment. The two-stage procedure which is considered the treatment of choice in patients who are medically stable^9,30,34,35^ and recommended when the infective microorganism is unknown^3^, has been commonly associated with successful eradication of infection in several case series^34,36,37^. However, this procedure frequently results in significant functional impairment^38^, given the requirement for two major surgical procedures. It is also costly for the healthcare system. The hospital cost of two-stage revision for the treatment of an infected shoulder arthroplasty is about two times higher than the cost of a primary shoulder arthroplasty^39^. The use of the one-stage revision strategy for managing PJI of the shoulder is becoming increasingly popular, though it has not gained the same level of momentum as with the treatment of PJI following hip arthroplasty. The main advantage of this procedure is that only one surgery is required, therefore it is associated with shorter hospital stay and generally shorter antibiotic duration^13,30,32^. The one-stage procedure is also associated with less tissue destruction and dissection, less patient anxiety, has the potential for better functional outcomes, and is associated with cost-benefits compared with the two-stage strategy^4,13,32,40^. Given the limited opportunities for further antibiotic therapy, the one-stage revision has commonly been adopted in select cases where the causative organisms have been identified pre-operatively^41^. Functional outcomes for one-stage revision have also been suggested to be dependent on the integrity of the rotator cuff muscles and type of prosthesis used^41^. Given the abundance of literature showing *C. acnes* as the common organism responsible for PJIs of the shoulder which is consistent with our findings, the potential to use the one-stage in a less selective manner is a possibility. Indeed, *C. acnes* is a gram-positive anaerobic bacillus which can be found in high concentrations in the acromion^42^ and can take up to four weeks to manifest as positive cultures^43^. PJI of the shoulder is associated with significant burden to the patient clinically and socioeconomically^5^ and also a challenge to the surgeon^7,44^. It also presents a huge financial burden to health systems^5,39^ and therefore a greater need to optimise resources. Indeed, previous reports suggest that shoulder PJIs have higher morbidity and costs compared with PJIs of other joints^45,46^. The current findings provide supportive evidence that the one-stage may be a potentially more attractive option for managing PJIs of the shoulders.

### Strengths and limitations

In addition to the several strengths enumerated above, our review employed a comprehensive search of several databases which yielded a large number of eligible studies compared to previous reviews on the topic. This ensured a more reliable comparison of the effectiveness of the two revision strategies in more detail than ever before. A detailed assessment of the methodological quality of the included studies was conducted using a well-established validated instrument. We adopted robust meta-analytic approaches which took into account the low event rates of the majority of the studies and heterogeneity between contributing studies. Other approaches included reporting of prediction intervals, comparison of re-infection rates among several clinically relevant characteristics, quantification of heterogeneity between studies and exploration of potential sources of bias. Our analyses showed no statistical evidence of publication bias and substantial heterogeneity between contributing studies for both revision strategies.

Several limitations deserve consideration. There was a comparatively smaller number of one-stage studies and limited data on the secondary clinical outcomes, which precluded the ability to robustly compare the two revision strategies head-to-head. The sparse data also precluded detailed subgroup analyses by relevant characteristics which could have influenced the outcomes such as sex, indication for primary procedure, co-morbidities, type of infecting organism, type of implant, and antibiotic duration. We contacted several authors to provide additional data but received only one response. Given the nature of data reported by contributing studies, we were unable to distinguish between cases of anatomic TSA versus RSA, revision versus primary shoulder arthroplasty, as well as recurrent versus new infections. Given the limitations of aggregate published data, these findings should be interpreted with caution. However, the current findings are both timely and relevant and highlight the one-stage revision strategy as an equally or more effective option for treating PJI of the shoulder. In the absence of a carefully designed RCT powered for re-infection outcomes to robustly compare the effectiveness of the two revision strategies, access to individual level data from published studies may help to confirm the present findings. Our group has recently successfully employed this approach to compare the effectiveness of the one- and two-stage revision strategies for the management of PJI of the hip^47^. Finally, there is a potential that data from national joint registries may be extremely useful in answering these questions, given their large-scale nature and cohort designs. However, no studies using registry data were eligible for the current review.

In conclusion, new evidence suggests the one-stage revision strategy is at least equally as effective as the two-stage revision strategy in controlling infection and improving function and pain as well noninfection complication rates in PJI of the shoulder following joint arthroplasty.

## Electronic supplementary material

Supplementary Material

## Data Availability

All data analysed during this study are available on reasonable request to the corresponding author.
