# Supplementary material for: One- and two-stage surgical revision of infected shoulder prostheses following arthroplasty surgery: A systematic review and meta-analysis
Source: Sci Rep. 2019 Jan 18;9:232. doi: 10.1038/s41598-018-36313-3 (PMC6338765; doi:10.1038/s41598-018-36313-3)
Supplement: Supplementary file 1 — Supplementary Material [file 41598_2018_36313_MOESM1_ESM.pdf]

One- and two-stage surgical revision of infected shoulder prostheses following arthroplasty surgery: A systematic review and meta-analysis

Running title: One- and two-stage surgical revision of infected shoulder prostheses

Setor K. Kunutsor[1,2*], Vikki Wylde[1,2], Andrew D. Beswick[2], Michael R. Whitehouse[1,2], Ashley W. Blom[1,2]

[1] National Institute for Health Research Bristol Biomedical Research Centre, University Hospitals Bristol NHS Foundation Trust and University of Bristol, Bristol, UK

[2]Translational Health Sciences, Bristol Medical School, Musculoskeletal Research Unit, University of Bristol, Learning & Research Building (Level 1), Southmead Hospital, Bristol, BS10 5NB, UK

Supplementary Material

**Appendix 1**	PRISMA checklist
**Appendix 2**	MOOSE checklist
**Appendix 3**	Literature search strategy
**Appendix 4**	Reference list of studies included in review
**Appendix 5**	Rates of re-infection in patients treated by two-stage revision, grouped according to study and population level characteristics

Appendix 1. PRISMA checklist

Section/topic	Item No	Checklist item	Reported on page No
**Title**			
Title	1	Identify the report as a systematic review, meta-analysis, or both	1
**Abstract**			
Structured summary	2	Provide a structured summary including, as applicable, background, objectives, data sources, study eligibility criteria, participants, interventions, study appraisal and synthesis methods, results, limitations, conclusions and implications of key findings, systematic review registration number	2
**Introduction**			
Rationale	3	Describe the rationale for the review in the context of what is already known	5-6
Objectives	4	Provide an explicit statement of questions being addressed with reference to participants, interventions, comparisons, outcomes, and study design (PICOS)	5
**Methods**			
Protocol and registration	5	Indicate if a review protocol exists, if and where it can be accessed (such as web address), and, if available, provide registration information including registration number	2
Eligibility criteria	6	Specify study characteristics (such as PICOS, length of follow-up) and report characteristics (such as years considered, language, publication status) used as criteria for eligibility, giving rationale	6
Information sources	7	Describe all information sources (such as databases with dates of coverage, contact with study authors to identify additional studies) in the search and date last searched	6
Search	8	Present full electronic search strategy for at least one database, including any limits used, such that it could be repeated	Appendix 3
Study selection	9	State the process for selecting studies (that is, screening, eligibility, included in systematic review, and, if applicable, included in the meta-analysis)	6-7
Data collection process	10	Describe method of data extraction from reports (such as piloted forms, independently, in duplicate) and any processes for obtaining and confirming data from investigators	6-7
Data items	11	List and define all variables for which data were sought (such as PICOS, funding sources) and any assumptions and simplifications made	6-7
Risk of bias in individual studies	12	Describe methods used for assessing risk of bias of individual studies (including specification of whether this was done at the study or outcome level), and how this information is to be used in any data synthesis	7-8
Summary measures	13	State the principal summary measures (such as risk ratio, difference in means).	7-8
Synthesis of results	14	Describe the methods of handling data and combining results of studies, if done, including measures of consistency (such as I^2 statistic) for each meta-analysis	7-8
Risk of bias across studies	15	Specify any assessment of risk of bias that may affect the cumulative evidence (such as publication bias, selective reporting within studies)	7-8
Additional analyses	16	Describe methods of additional analyses (such as sensitivity or subgroup analyses, meta-regression), if done, indicating which were pre-specified	7-8
**Results**			
Study selection	17	Give numbers of studies screened, assessed for eligibility, and included in the review, with reasons for exclusions at each stage, ideally with a flow diagram	8 and Fig. 1
Study characteristics	18	For each study, present characteristics for which data were extracted (such as study size, PICOS, follow-up period) and provide the citations	8-9, Table 2
Risk of bias within studies	19	Present data on risk of bias of each study and, if available, any outcome-level assessment (see item 12).	9-10, Table 2
Results of individual studies	20	For all outcomes considered (benefits or harms), present for each study (a) simple summary data for each intervention group and (b) effect estimates and confidence intervals, ideally with a forest plot	9-10, Figs. 2-3
Synthesis of results	21	Present results of each meta-analysis done, including confidence intervals and measures of consistency	9-10, Figs. 2-3

Section/topic	Item No	Checklist item	Reported on page No
Risk of bias across studies	22	Present results of any assessment of risk of bias across studies (see item 15)	9-10
Additional analysis	23	Give results of additional analyses, if done (such as sensitivity or subgroup analyses, meta-regression) (see item 16)	9-10, Appendix 5
**Discussion**			
Summary of evidence	24	Summarise the main findings including the strength of evidence for each main outcome; consider their relevance to key groups (such as health care providers, users, and policy makers)	11
Limitations	25	Discuss limitations at study and outcome level (such as risk of bias), and at review level (such as incomplete retrieval of identified research, reporting bias)	14
Conclusions	26	Provide a general interpretation of the results in the context of other evidence, and implications for future research	12-13
**Funding**			
Funding	27	Describe sources of funding for the systematic review and other support (such as supply of data) and role of funders for the systematic review	15-16

Appendix 2. MOOSE checklist

One- and two-stage surgical revision of infected shoulder prostheses following arthroplasty surgery: A systematic review and meta-analysis

Criteria		Brief description of how the criteria were handled in the review
**Reporting of background**		
√	Problem definition	Periprosthetic joint infection (PJI) of the shoulder is less frequent and uncommon compared to PJI of the knee and hip; however, it is a catastrophic complication. Two main treatment options exist and which include one-stage or two-stage revision. Several studies have been carried out on this topic, but the best treatment option is currently uncertain. In this context, we have carried out a systematic review and meta-analysis of longitudinal studies to compare the effectiveness of the one- and two-stage revision strategies in terms of re-infection outcomes and other clinical outcomes.
√	Hypothesis statement	There is no difference in re-infection outcomes after one-stage or two-stage revision surgery for shoulder PJI
√	Description of study outcomes	Rates of re-infection (number of re-infections and or recurrence of infection); Function as measured by (i) Constant-Murley score (CMS), American Shoulder and Elbow (ASES) Shoulder Assessment scores, Simple Shoulder Test (SST), Disabilities of the Arm, UCLA score (function component), Shoulder and Hand score (DASH), Penn Shoulder Score (function component), forward elevation, forward flexion, abduction, external rotation, and range of motion]; (i) pain [as measured by pain scores, visual analogue scores (VAS), Penn Shoulder Score (pain component), University of California Los Angeles (UCLA) score (pain component)]; and (iii) satisfaction [as measured by Penn Shoulder Score (satisfaction component)].
√	Type of exposure	One-stage and two-stage surgical revision of infected shoulder prosthesis
√	Type of study designs used	Longitudinal studies (retrospective, prospective, or randomised controlled trials)
√	Study population	Patients treated exclusively by one-stage or two-stage revision
**Reporting of search strategy should include**		
√	Qualifications of searchers	Setor Kunutsor, PhD; Andrew Beswick, BSc; Vikki Wylde, PhD
√	Search strategy, including time period included in the synthesis and keywords	Time period: From inception to 10 February 2018.<br>The detailed search strategy can be found in Appendix 3.
√	Databases and registries searched	MEDLINE, EMBASE, Web of Science, and Cochrane databases
√	Search software used, name and version, including special features	OvidSP was used to search EMBASE and MEDLINE<br>EndNote used to manage references
√	Use of hand searching	We searched bibliographies of retrieved papers
√	List of citations located and those excluded, including justifications	Details of the literature search process are outlined in the flow chart. The citation list for excluded studies are available on request.
√	Method of addressing articles published in languages other than English	We placed no restrictions on language
√	Method of handling abstracts and unpublished studies	We contacted investigators for unpublished data on the topic.
√	Description of any contact with authors	We contacted authors of studies that did not provide adequate data for analysis
**Reporting of methods should include**		
√	Description of relevance or appropriateness of studies assembled for assessing the hypothesis to be tested	Detailed inclusion and exclusion criteria are described in the Methods section.
√	Rationale for the selection and coding of data	Data extracted from each of the studies were relevant to the population characteristics, study design, exposure, and outcome.
√	Assessment of confounding	We included only studies where populations were unselected
√	Assessment of study quality, including blinding of quality assessors; stratification or regression on possible predictors of study results	Study quality was assessed based on the Methodological Index for Non-Randomised Studies (MINORS), a validated instrument which is designed for assessment of methodological quality of non-randomised studies in surgery
√	Assessment of heterogeneity	Heterogeneity of the studies was quantified with I^2 statistic that provides the

		relative amount of variance of the summary effect due to the between-study heterogeneity and explored using meta-regression and stratified analyses
√	Description of statistical methods in sufficient detail to be replicated	Description of methods of meta-analyses, sensitivity analyses, meta-regression and assessment of publication bias are detailed in the methods. We performed random effects meta-analysis with Stata 14.
√	Provision of appropriate tables and graphics	Tables 1-2; Appendix 5; Figures 1-3
**Reporting of results should include**		
√	Graph summarizing individual study estimates and overall estimate	Figs. 2-3
√	Table giving descriptive information for each study included	Appendix 5
√	Results of sensitivity testing	Sensitivity analysis was conducted to assess the influence of some large studies and low quality studies on the pooled estimate. This was done by omitting such studies and calculating a pooled estimate for the remainder of the studies
√	Indication of statistical uncertainty of findings	95% confidence intervals were presented with all summary estimates, I^2 values and results of sensitivity analyses
**Reporting of discussion should include**		
√	Quantitative assessment of bias	Sensitivity analyses indicate heterogeneity in strengths of the association due to most common biases in observational studies. The systematic review is limited in scope, as it involves published data. Individual participant data (IPD) meta-analysis is needed. Limitations have been discussed.
√	Justification for exclusion	All studies were excluded based on the pre-defined inclusion criteria in methods section.
√	Assessment of quality of included studies	Brief discussion included in 'Methods' section
**Reporting of conclusions should include**		
√	Consideration of alternative explanations for observed results	Discussion
√	Generalization of the conclusions	Discussed in the context of the results.
√	Guidelines for future research	We recommend evidence from a carefully designed randomised clinical trial or IPD meta-analysis
√	Disclosure of funding source	In "Source of Funding" section

Appendix 3. Literature search strategy

Relevant studies, published before 10 February 2018 (date last searched), were identified through electronic searches not limited to the English language using MEDLINE, EMBASE, Web of Science, and Cochrane databases. Electronic searches were supplemented by scanning reference lists of articles identified for all relevant studies (including review articles), by hand searching of relevant journals and by correspondence with study investigators. The computer-based searches combined search terms related to shoulder replacement, periprosthetic joint infection, and revision with focus on one- and two stage surgeries.

1 infect$.mp. or Infection/ (2484055)
2 shoulder.mp. (87247)
3 rotator cuff.mp. or Rotator Cuff/ (13736)
4 2 or 3 (90108)
5 one-stage.mp. (13288)
6 two-stage.mp. (27557)
7 revis$.mp. (220006)
8 5 or 6 or 7 (257280)
9 1 and 4 and 8 (652)
34 22 and 23 and 33 (7306)
35 limit 34 to humans (7197)

Each part was specifically translated for searching the other databases (EMBASE, Web of Science, and Cochrane databases)

Appendix 4. Reference list of included studies

1. Coste JS, Reig S, Trojani C, Berg M, Walch G, Boileau P. The management of infection in arthroplasty of the shoulder. *J Bone Joint Surg Br.* 2004;86(1):65-69.

2. Beekman PD, Katusic D, Berghs BM, Karelse A, De Wilde L. One-stage revision for patients with a chronically infected reverse total shoulder replacement. *J Bone Joint Surg Br.* 2010;92(6):817-822.

3. Grosso MJ, Sabesan VJ, Ho JC, Ricchetti ET, Iannotti JP. Reinfection rates after 1-stage revision shoulder arthroplasty for patients with unexpected positive intraoperative cultures. *J Shoulder Elbow Surg.* 2012;21(6):754-758.

4. Amaravathi RS, Kany J, Melet M, et al. Analysis of infection in shoulder arthroplasty: a multicentre study. *European Journal of Orthopaedic Surgery & Traumatology.* 2012;22(2):145-150.

5. Klatte TO, Junghans K, Al-Khateeb H, et al. Single-stage revision for peri-prosthetic shoulder infection: outcomes and results. *Bone Joint J.* 2013;95-B(3):391-395.

6. Middernacht B, Van Tongel A, De Wilde L. Reversed Revised: What to do when it goes wrong? *Acta Orthop Belg.* 2014;80(3):314-321.

7. Jacquot A, Sirveaux F, Roche O, Favard L, Clavert P, Mole D. Surgical management of the infected reversed shoulder arthroplasty: a French multicenter study of reoperation in 32 patients. *J Shoulder Elbow Surg.* 2015;24(11):1713-1722.

8. Stone GP, Clark RE, O'Brien KC, et al. Surgical management of periprosthetic shoulder infections. *J Shoulder Elbow Surg.* 2017;26(7):1222-1229.

9. Sperling JW, Kozak TK, Hanssen AD, Cofield RH. Infection after shoulder arthroplasty. *Clin Orthop.* 2001(382):206-216.

10. Seitz WH, Jr., Damacen H. Staged exchange arthroplasty for shoulder sepsis. *J Arthroplasty.* 2002;17(4 Suppl 1):36-40.

11. Jerosch J, Schneppenheim M. Management of infected shoulder replacement. *Arch Orthop Trauma Surg.* 2003;123(5):209-214.

12. Mileti J, Sperling JW, Cofield RH. Reimplantation of a shoulder arthroplasty after a previous infected arthroplasty. *J Shoulder Elbow Surg.* 2004;13(5):528-531.

13. Dines JS, Fealy S, Strauss EJ, et al. Outcomes analysis of revision total shoulder replacement. *J Bone Joint Surg Am.* 2006;88(7):1494-1500.

14. Strickland JP, Sperling JW, Cofield RH. The results of two-stage re-implantation for infected shoulder replacement. *J Bone Joint Surg Br.* 2008;90(4):460-465.

15. Kelly JD, 2nd, Hobgood ER. Positive culture rate in revision shoulder arthroplasty. *Clin Orthop.* 2009;467(9):2343-2348.

16. Dodson CC, Craig EV, Cordasco FA, et al. Propionibacterium acnes infection after shoulder arthroplasty: a diagnostic challenge. *J Shoulder Elbow Surg.* 2010;19(2):303-307.

17. Hattrup SJ, Renfree KJ. Two-stage shoulder reconstruction for active glenohumeral sepsis. *Orthopedics.* 2010;33(1):20.

18. Stine IA, Lee B, Zalavras CG, Hatch G, 3rd, Itamura JM. Management of chronic shoulder infections utilizing a fixed articulating antibiotic-loaded spacer. *J Shoulder Elbow Surg.* 2010;19(5):739-748.

19. Jawa A, Shi L, O'Brien T, et al. Prosthesis of antibiotic-loaded acrylic cement (PROSTALAC) use for the treatment of infection after shoulder arthroplasty. *J Bone Joint Surg Am.* 2011;93(21):2001-2009.

20. Sabesan VJ, Ho JC, Kovacevic D, Iannotti JP. Two-stage reimplantation for treating prosthetic shoulder infections. *Clin Orthop.* 2011;469(9):2538-2543.

21. Weber P, Utzschneider S, Sadoghi P, Andress HJ, Jansson V, Muller PE. Management of the infected shoulder prosthesis: a retrospective analysis and review of the literature. *Int Orthop.* 2011;35(3):365-373.

22. Romano CL, Borens O, Monti L, Meani E, Stuyck J. What treatment for periprosthetic shoulder infection? Results from a multicentre retrospective series. *Int Orthop.* 2012;36(5):1011-1017.

23. Achermann Y, Sahin F, Schwyzer HK, Kolling C, Wust J, Vogt M. Characteristics and outcome of 16 periprosthetic shoulder joint infections. *Infection.* 2013;41(3):613-620.

24. Ghijselings S, Stuyck J, Debeer P. Surgical treatment algorithm for infected shoulder arthroplasty: a retrospective analysis of 17 cases. *Acta Orthop Belg.* 2013;79(6):626-635.

25. Ortmaier R, Resch H, Hitzl W, Mayer M, Stundner O, Tauber M. Treatment strategies for infection after reverse shoulder arthroplasty. *Eur J Orthop Surg Traumatol.* 2014;24(5):723-731.

26. Zhang AL, Feeley BT, Schwartz BS, Chung TT, Ma CB. Management of deep postoperative shoulder infections: is there a role for open biopsy during staged treatment? *J Shoulder Elbow Surg.* 2015;24(1):e15-20.

27. Assenmacher AT, Alentorn-Geli E, Dennison T, et al. Two-stage reimplantation for the treatment of deep infection after shoulder arthroplasty. *J Shoulder Elbow Surg.* 2017;26(11):1978-1983.

28. Buchalter DB, Mahure SA, Mollon B, Yu S, Kwon YW, Zuckerman JD. Two-stage revision for infected shoulder arthroplasty. *J Shoulder Elbow Surg.* 2017;26(6):939-947.

29. Lee SH, Kim SJ, Kook SH, Kim JW. Two-stage revision of infected shoulder arthroplasty using prosthesis of antibiotic-loaded acrylic cement: minimum three-year follow-up. *Int Orthop.* 2017.

30. Grubhofer F, Imam MM, Wieser K, Achermann Y, Meyer DC, Gerber C. Staged Revision With Antibiotic Spacers for Shoulder Prosthetic Joint Infections Yields High Infection Control. *Clin Orthop.* 2018;476(1):146-152.

Appendix 5. Rates of re-infection in patients treated by two-stage revision, grouped according to study and population level characteristics

Subgroup	Re-infections / Participants or shoulders†	Rate of re-infection (95% CI)	*P*-value*
**Location**			
Europe	22 / 131	13.40 (3.00, 27.70)	.84
North America	35 / 208	12.40 (6.30, 19.60)	
Asia	0 / 12	0.00 (0.00, 24.20)	
**Average age (years)**			
≥ 65	11 / 97	6.70 (0.80, 16.00)	.72
< 65	20 / 124	12.40 (3.80, 23.70)	
**Follow-up (years)**			
≥ 4	16 / 122	9.00 (3.20, 16.30)	.78
< 4	23 / 139	11.90 (3.60, 23.10)	
**No. of participants**			
≥ 15	33 / 212	14.20 (7.30, 22.50)	.89
> 15	24 / 139	10.10 (2.40, 20.60)	
**Quality score**			
≥ 11	23 / 174	10.40 (4.00, 18.50)	.58
> 11	34 / 177	12.20 (4.40, 22.20)	

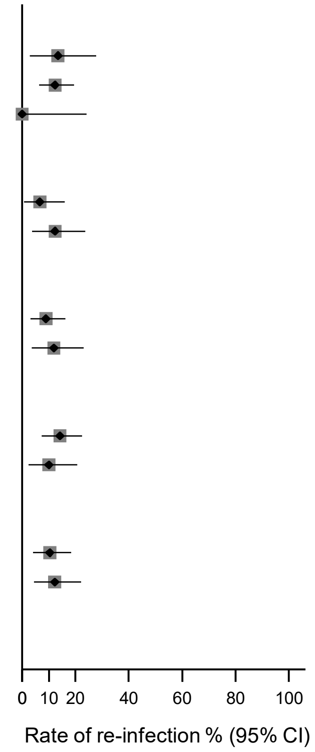

CI, confidence interval (bars); *, *P*-value for meta-regression; †, number of infections and participants do not add up to the overall total because of missing data
